# Person‐centred sexual and reproductive health: A call for standardized measurement

**DOI:** 10.1111/hex.13781

**Published:** 2023-05-25

**Authors:** Patience A. Afulani, Michelle K. Nakphong, May Sudhinaraset

**Affiliations:** ^1^ Departments of Epidemiology and Biostatistics and Obstetrics, Gynecology, and Reproductive Sciences, School of Medicine University of California, San Francisco San Francisco California USA; ^2^ Department of Community Health Sciences, Jonathan and Karin Fielding School of Public Health University of California, Los Angeles Los Angeles California USA

**Keywords:** experience of care, measurement, person‐centred care, respectful care, sexual and reproductive health and rights

## Abstract

Person‐centred sexual and reproductive health (PCSRH) care refers to care that is respectful of and responsive to people's preferences, needs, and values, and which empowers them to take charge of their own sexual and reproductive health (SRH). It is an important indicator of SRH rights and quality of care. Despite the recognition of the importance of PCSRH, there is a gap in standardized measurement in some SRH services, as well as a lack of guidance on how similar person‐centred care measures could be applied across the SRH continuum. Drawing on validated scales for measuring person‐centred family planning, abortion, prenatal and intrapartum care, we propose a set of items that could be validated in future studies to measure PCSRH in a standardized way. A standardized approach to measurement will help highlight gaps across services and facilitate efforts to improve person‐centred care across the SRH continuum.

**Patient or Public Contribution:**

This viewpoint is based on a review of validated scales that were developed through expert reviews and cognitive interviews with services users and providers across the different SRH services. They provided feedback on the relevance, clarity, and comprehensiveness of the items in each scale.

## 1 INTRODUCTION

Access to respectful, person‐centred sexual and reproductive health (PCSRH) services is a fundamental human right and critical to ensuring equitable care globally. The achievement of PCSRH is dependent on the realization of sexual and reproductive health (SRH) rights—the rights of individuals to make decisions governing their bodies and to access services that support those rights and includes among other things the rights of all individuals to bodily integrity, privacy and personal autonomy.^1^ While there has been a growing recognition of the need for respectful, person‐centred care, there has been a critical lack of consensus on how to measure this across multiple services. A standardized approach to measurement is useful for highlighting common gaps across services to facilitate efforts to improve person‐centred care across the SRH continuum. This paper puts forth potential measures for future researchers, implementers and initiatives.

## 2 WHAT IS PCSRH?

We define PCSRH as care that is respectful of and responsive to people's preferences, needs and values, and which empowers people to take charge of their own SRH. This builds on the definition of patient‐centred care from the US Institute of Medicine (National Academy of Medicine since 2015), which highlights person‐centred care as a key domain of quality of care.^2^ This last part of the definition draws on the World Health Organization definition of integrated people‐centred health systems, which emphasizes ‘empowering people to take charge of their own health rather than being passive recipients of services’ and ‘putting people and communities, not diseases, at the centre of health systems’.^3^ PCSRH is thus care that promotes reproductive autonomy, is free of reproductive coercion, elevates individual's decision‐making and is supportive and empowering. Domains of PCSRH include dignity, communication, autonomy, privacy, confidentiality, social support, supportive care, trust and the health facility environment.^4^, ^5^

We have chosen to use the term ‘person‐centred’ over ‘patient‐centred’ as per discussions that ‘patient’ tends to objectify and reduce the person to a mere recipient of medical services, or to ‘one who is acted on’.^6^ Person‐centred care highlights the importance of ‘knowing the person behind the patient–as a human being with reason, will, feelings and needs—to engage the person as an active partner in their care and treatment’.^6^ The term ‘person‐centred’ is also inclusive of family and significant others who are often co‐decision‐makers in whether and how people seek and use care. Although the singular terminology highlights the specific needs and preferences of the care seeker, people‐centred care is also an appropriate terminology to capture these social networks. In addition, people‐centred care as used in the WHO terminology highlights the responsiveness of the health system to the community, which is inclusive of individual needs.^3^

Regardless of terminology, this concept highlights respect for individuals of all backgrounds and identities, including treating people with dignity, respecting their privacy, keeping their health information confidential, providing sufficient information for people to make informed decisions about their care, providing emotional and social support and valuing their individual preferences and diversity—emphasizing the interpersonal and experience dimensions of care.^2^, ^4^, ^7^ For example, person‐centred decision‐making is an approach to shared decision‐making predicated on the need for clinicians to understand and respect the patient as a person to fully engage with the patient's experience of illness and participation in their treatment. This then allows clinicians to provide supportive care that respects a person's autonomy.^8^

## 3 A LIFE COURSE PERSPECTIVE OF SRH

A life course perspective moves beyond the traditional understanding of reproductive health, which tends to focus only on contemporary risk factors and the independent nature of these risks.^9^ The life course perspective, on the other hand, considers the entire life span of an individual, the continuity of reproductive health care and the temporal order of exposures.^9^ Past studies have found that early life exposures are linked to later reproductive health outcomes; and women's reproductive health is linked to previous generations and predictive of later chronic conditions, such as cardiovascular diseases and cancers.^10^, ^11^ Based on the Guttmacher‐Lancet Commission report, the continuum of SRH care includes contraceptive services; counselling and care for sexual health and wellbeing; prevention and treatment of HIV/AIDS; care for other sexually transmitted infections (STIs); comprehensive sexuality education; safe abortion care; prevention, detection and counselling for gender‐based violence; maternal and newborn care; and prevention, detection and treatment of infertility and cervical cancer.^1^ These SRH services span most phases of an individual's life course, hence the need for a life course perspective to SRH.

## 4 BENEFITS OF PCSRH

Person‐centred care, and by extension PCSRH, has direct and indirect effects on health outcomes by influencing health‐seeking behaviour, self‐efficacy, patient engagement, timely and appropriate care, safety and improved psychosocial health.^12^, ^13^, ^14^ For example, mistreatment of women during childbirth deters women from giving birth in health facilities,^15^, ^16^, ^17^, ^18^ while providing continuous support during childbirth is associated with shorter labour, increased likelihood of spontaneous vaginal delivery, lower anxiety, better coping with pain and increased rates of breastfeeding initiation.^19^ Information‐sharing and positive interpersonal interactions can increase the adoption and continuation of modern family planning methods.^20^, ^21^ Person‐centred care is critical across the life course as quality of care experiences early in life may shape future health outcomes, as well as people's understanding of, expectations and decisions to seek timely SRH care. Particularly for people from marginalized groups or people with marginalized identities, who are more likely to be mistreated or neglected in healthcare settings, person‐centred care calls attention to the equitable delivery of care.^22^, ^23^

### 4.1 Measurement and state of PCSRH

Several measures have been developed and validated across various settings to measure PCSRH including family planning services, abortion services, prenatal care and intrapartum care.^24^, ^25^, ^26^, ^27^, ^28^, ^29^, ^30^, ^31^, ^32^, ^33^, ^34^, ^35^, ^36^, ^37^ Other measures focus on specific aspects of person‐centred care, including the interpersonal quality of contraceptive counselling^35^; and process domain measures such as respectful care, method selection, effective use of selected methods and continuity of contraceptive use and care and shared‐decision making.^34^, ^38^, ^39^ These measures demonstrate valid and reliable ways of measuring PCSRH and highlight the interrelated domains of person‐centred care including respect, dignity, autonomy, communication, supportive care and health facility environment. There are, however, no standardized person‐centred care measures that can be applied across several SRH services.

While there are other examples of different measures of PCSRH, we use the person‐centred maternity care (PCMC),^24^, ^25^, ^31^ person‐centred prenatal care (PCPC),^28^, ^30^ person‐centred family planning care (PCFP)^26^ and person‐centred abortion care (PCAC)^29^ scales as examples of how standardized PCSRH measures can be used to compare across contexts and SRH outcomes. These scales were all rigorously developed and validated using standard procedures for scale development including literature reviews, expert reviews, cognitive interviews, surveys and psychometric analysis—with evidence of high validity and reliability—which are described in detail in the validation manuscripts.^24^, ^25^, ^26^, ^27^, ^28^, ^29^, ^30^, ^31^ Each scale has items and subscales capturing the key domains of person‐centred care (Table 1). Although there are some differences in the type and number of items across these scales, scores on all the scales can be standardized to range from 0 to 100, with higher scores indicative of more person‐centred care. For example, for maternity care, the total standardized scores for surveys conducted in Kenya, Ghana and India were all below 70 out of a total of 100.^40^ Scores on the family planning scales were at 70 for Kenya and 87 for India,^26^ while for abortion, scores were in the range of 83–86 for medication and surgical abortion, respectively.^29^

**Table 1:** Summary of person‐centred care scales across family planning, abortion, and maternity care.^a^

Scale	Context	# of items	Subdomains
Person‐centred family planning scale	Kenya	20	Autonomy and respect (14 items) Health facility climate (6 items)
	India	22	Autonomy and respect (17 items) Health facility climate (5 items)
Person‐centred abortion scale	Kenya	*Medication*: 23 items *Surgical*: 24 items	Communication and autonomy (9 items) Respectful and supportive care (14 items for medication abortion, 15 items for surgical abortion)
Person‐centred prenatal care scale	California	34	Dignity and respect (14 items) Communication and autonomy (10 items) Responsive and supportive care (10 items)
	Kenya^b^	18	
Person‐centred maternity scale^c^	India	27	Dignity and respect (6 items) Communication and autonomy (9 items) Supportive care (12 items)
	Kenya (urban & rural combined)	30	Dignity and respect (6 items) Communication and autonomy (9 items) Supportive care (15 items)
	Ghana	30	Dignity and respect (6 items) Communication & autonomy (9 items) Supportive care (15 items)
	California	35	Dignity and respect (10 items) Communication and autonomy (14 items) Responsive and supportive care (11 items)

^a^ Details in Afulani and colleagues.^24^, ^25^, ^26^, ^27^, ^28^, ^29^, ^30^, ^31^, ^40^

^b^ This is an experience of care index that includes several generic person‐centred care questions as well as questions specific to antenatal care procedures. The items were not based on a formal process of scale development and validation.

^c^ For intrapartum care.

## 5 GAPS IN PCSRH

The domain with the most prominent gaps across settings and SRH outcomes was Communication and Autonomy. Standardized PCMC subscale scores (range of 0–100) for communication and autonomy were below 60 for Kenya, Ghana and India. Similarly, for abortion care, communication and autonomy scores were significantly lower compared to the respectful and supportive care domains. These gaps are reflected in individual items in the scales. For instance, over 70% of women surveyed on their maternity care experiences in Ghana, Kenya and India reported providers never introduced themselves.^40^ Similarly, high proportions of women surveyed in Kenya and India on their family planning and abortion experiences reported providers did not introduce themselves.^26^, ^29^ Additionally, more than one out of three women surveyed in Kenya, Ghana and India reported they were not asked for consent for examinations and procedures during childbirth.^40^ These findings highlight gaps in person‐centred care at various stages of the SRH continuum that need to be addressed.

## 6 PERSON‐CENTRED CARE ITEMS CAN BE MEASURED ACROSS THE SRH CONTINUUM

Common items in the scales for family planning, abortion, prenatal and intrapartum care highlight the potential for a common person‐centred care scale across SRH outcomes. Thus, to identify key person‐centred care items that can be measured across the SRH continuum, we examined the individual items in each of the scales for items that were common across all the scales (Supporting Information: Appendix 1). Eleven items were common across the PCFP, PCMC, PCPC and PCAC scales. However, there were notably missing key indicators due to some items having fallen out during psychometric analysis for the abortion or family scales, but which we identified as gap areas for improvement. Thus, we decided to identify items that were common on at least 3 scales which yielded 25 items (Box 1). A review of these 25 items shows that there are aspects of person‐centred care that are not limited to any phase of care. For example, indicators that tap into the construct of person‐centred decision‐making (e.g., involved in care, explaining exams) were common across scales. Based on this, we propose these items to serve as the basis for a common person‐centred care scale across SRH outcomes that can be tested in future studies. Suggested question wordings for these items are shown in Supporting Information: Appendix 2.

BOX 1 **Person‐centred care items that can be measured across the SRH continuum**

*Dignity and respect*

1. **Treated with respect**
2. **Information confidentiality**
3. **Providers friendly**
4. Visual privacy
5. Verbal abuse
6. Physical abuse
7. Discrimination/treated differently
8. Providers cared
*Communication and autonomy*
9. **Providers introduce self**
10. **Called appropriately**
11. **Involved in care**
12. **Explain exams**
13. Explain medicines
14. Consent before exams/procedures
15. Able to ask questions
16. Language you understood
*Responsive and supportive care*
17. **Wait time**
18. **Took best care**
19. **Trust providers**
20. **Felt safe**
21. Bribes
22. Ask about feeling
23. Paid attention when help needed
24. Enough staff
25. Cleanliness

Notes: These are person‐centred care items found in at least 3 stages of the SRH continuum.

Bold items (first 3 to 4 in each domain) are common across the person‐centred care scales for abortion, family planning, prenatal care, and intrapartum care.^22^, ^23^, ^24^, ^25^, ^26^, ^27^, ^28^, ^29^, ^36^

## 7 LIMITATIONS OF MEASURES FOR PERSON‐CENTRED CARE ACROSS THE SRH CONTINUUM

We propose these measures as a starting point for future research on person‐centred care across the SRH continuum. However, we acknowledge there are limitations and suggest areas for future research. First, these measures were developed for services related to contraception, abortion, prenatal and intrapartum care. While there have been other person‐centred care measures for services such as HIV,^41^ there is relatively little on standardized person‐centred care measures for other services such as other STIs^42^; infertility and cervical cancer care^43^, ^44^; prevention, detection and counselling for gender‐based violence,^45^ especially in low‐ and middle‐income countries (LMICs). For example, while quality of care frameworks have been developed for gender‐based violence services, there are few standardized measures that operationalize people's experiences of care.^45^ The proposed items, therefore, do not capture the nuances of different SRH care experiences. For SRH outcomes for which there are no existing measures, these items only serve as a starting point and additional items are needed to capture experiences relevant to those outcomes.

Second, even for measures developed on family planning, abortion and maternity care services, there is a need to recognize the changing policy landscapes across services. For example, abortion services are constantly under threat, and in some parts of the world, like the United States, are becoming increasingly restrictive. Health facilities and providers, therefore, may need to be responsive and change protocols such as the increased use of telehealth to provide medication abortions.^46^ Person‐centred care measures for abortions may need to be reflective of the different approaches introduced, including examining issues of privacy and confidentiality in the era of telehealth services, or determining autonomy in decision‐making regarding abortion procedures in the context of state‐level restrictions.^47^

Further, while these measures are based on studies in LMICs and the United States, future research may want to explore more context‐specific indicators. To adapt and validate these measures in specific contexts, cognitive interviews are particularly important to understand how women make meaning from their specific experiences and environments.^48^ For example, in Kenya, expert reviewers and women who had recently given birth indicated that the health facility environment such as having water, electricity and enough staff were important aspects of their care experience; however, when these measures were fielded in a US setting, these measures became less salient. Instead, expert reviewers and women discussed a generally comfortable birth environment as critical to person‐centred care. While both are related to the overall health facility environment, there are differences in the ways that women describe the aspects that are most relevant to them. Related, even within specific contexts, population‐specific indicators may be needed to fully capture person‐centred care experiences that may be salient to certain groups. For example, for immigrant populations, nuanced indicators around language and translation services may be needed.

## 8 A CALL TO ACTION

A standardized approach to examining person‐centred care across the SRH spectrum is critical to highlighting areas of deficit and disparities across services and to ensuring equity for all individuals. Given that the same provider may be providing multiple SRH services, they could be trained on multiple services simultaneously. Further research is, however, needed to develop measures relevant to other aspects of SRH and across the life course, including person‐centred care for newborns and children's care, adolescent SRH, infertility, reproductive cancers and gender‐based violence. The common indicators across PCFP, PCMC, PCPC, and PCAC may serve as a starting point for measuring person‐centred care in these areas of SRH. Other areas of SRH that need attention are the treatment of family members and support persons during care, provider perspectives on person‐centred care, and the unique needs of special and marginalized populations, such as gender and sexual minorities, immigrants and other vulnerable populations. Further, standardizing person‐centred care measures across contexts and across the SRH continuum will help identify where and which services need the most attention in different settings. In this piece, we focused on PCSRH, because the items were pulled from SRH scales. The questions identified are, however, agnostic to the type of care encountered and could potentially be used at any stage of the life course. Future work examining how these items align with measures beyond SRH will facilitate their application beyond SRH. These measures should be included in routine national data collection systems for ongoing monitoring and intervention efforts. Most importantly, measurement should facilitate efforts to improve PCSRH and person‐centred care more broadly, as evidence‐based interventions to improve person‐centred care are urgently needed.

## CONFLICT OF INTEREST STATEMENT

The authors declare no conflicts of interest

## Supporting information

Supporting information.

Supporting information.

## Data Availability

Data sharing is not applicable to this article as no datasets were generated or analyzed during the current study.
