# Supplementary material for: Person‐centred sexual and reproductive health: A call for standardized measurement
Source: Health Expect. 2023 May 25;26(4):1384–90. doi: 10.1111/hex.13781 (PMC10349248; doi:10.1111/hex.13781)
Supplement: Supplementary file 1 — Supporting information. [file HEX-26--s001.docx]

**Appendix 1. Person-centered care items across different stages of the reproductive life course**

|  | **FAMILY PLANNING** | **ABORTION** | **PRENATAL CARE** | **MATERNITY CARE** |
| --- | --- | --- | --- | --- |
| **Dignity & Respect** | | | | |
| Treated with respect * | X | X | X | X |
| Friendly * | X | X | X | X |
| Visual privacy * |  | X | X | X |
| Information/Record confidentiality * | X | X | X | X |
| Privacy - knock |  |  |  | X |
| Verbal abuse * |  | X | X | X |
| Physical abuse * |  | X | X | X |
| Discrimination* |  | X | X | X |
| Family respected |  |  | X | X |
| Neglected |  |  | X | X |
| Knowledge/Experience valued |  |  | X | X |
| Cared* | X | X |  | X |
| Customs respected |  |  |  | X |
| **Communication & Autonomy** |  |  |  |  |
| Introduce self * | X | X | X | X |
| Called by name * | X | X | X | X |
| Involvement in care/decisions * | X | X | X | X |
| Consent to procedures * |  | X | X | X |
| Language you understood* | X | X |  | X |
| Explain exams & procedures * | X | X | X | X |
| Understood exams & procedures | X |  | X |  |
| Explain medicines * |  | X | X | X |
| Understood medicines | X |  | X |  |
| Checked understanding |  |  | X | X |
| Able to ask questions * | X |  | X | X |
| Asked if you had questions |  | X | X |  |
| Hold back asking questions |  |  | X | X |
| Felt heard/listened to |  |  | X | X |
| Felt informed/Enough information | X |  |  | X |
| Coercion |  |  | X | X |
| Birth plan/Birth preferences respected |  |  | X | X |
| Delivery position choice |  |  |  | X |
| Baby feeding choice respected |  |  |  | X |
| Explain baby procedures |  |  |  | X |
| **Responsive & Supportive Care** |  |  |  |  |
| Trust providers * | X | X | X | X |
| Wait time/Time to care * | X | X | X | X |
| Time with provider |  |  | X |  |
| Attention when needed help/Paid attention * | X | X |  | X |
| Took best care * | X | X | X | X |
| Care about you as a person | X |  |  |  |
| Information showed they cared |  |  | X |  |
| Talk/Ask about feeling * | X | X |  | X |
| Ask about pain | X | X |  |  |
| Control pain/Pain management |  |  |  | X |
| Pain medication given | X | X |  |  |
| Support anxiety | X |  |  | K |
| Allowed support person/companion during labor or delivery ^1^ | X |  |  | X |
| Bribes * | X | X | X | X |
| Emotional well-being |  |  | X | X |
| Resources for emotional well-being |  |  | X |  |
| Preferred clinic |  |  | X |  |
| Preferred provider |  |  | X |  |
| Believed about pain |  |  |  | X |
| Support for baby feeding |  |  |  | X |
| **Health Facility Environment** |  |  |  |  |
| Felt Safe * | X | X | X | X |
| Enough staff * | X | X |  | X |
| Crowded | X |  |  | X |
| Clean facility * | X |  | X | X |
| Clean bathroom | X |  |  |  |
| Water | X |  |  | K |
| Electricity | X |  |  | K |
| Comfortable birth environment |  |  |  | X |

Notes

X Indicates present in at least one version of the scale for that SRH service

* Indicates PCC item in at least 3 stages of the life course

^1^ For maternity care, two separate questions ask whether support persons were allowed during labor and delivery, respectively.
