# Supplementary material for: Person‐centred sexual and reproductive health: A call for standardized measurement
Source: Health Expect. 2023 May 25;26(4):1384–90. doi: 10.1111/hex.13781 (PMC10349248; doi:10.1111/hex.13781)
Supplement: Supplementary file 2 — Supporting information. [file HEX-26--s002.docx]

**Appendix 2. Question wording for items recommended for measuring Person-centered sexual and reproductive health**

| Label | Question wording |
| --- | --- |
| **Dignity & Respect** | |
| Treated with respect | Did the doctors, nurses, or other staff at the facility treat with you respect? |
| Information confidentiality | Do you feel like your health information was or will be kept confidential at this facility? |
| Friendly | Did the doctors, nurses, and other staff at the facility treat you in a friendly manner? |
| Visual Privacy | During examinations, were you covered up with a cloth or blanket or screened with a curtain so that you did not feel exposed? |
| Verbal abuse | Do you feel the doctors, nurses, or other health providers shouted at you, scolded you, insulted, threatened, or talked to you rudely? |
| Physical abuse | Did you feel like you were treated roughly like pushed, beaten, slapped, pinched, physically restrained, or gagged? |
| Discrimination/Treated differently | During your time in the health facility, would you say you were treated differently because of any personal attribute…like your age, marital status, number of children, your education, wealth, your connections with the facility, or something like that? |
| Cared | Did the doctors, nurses, and other staff at the facility show they cared for you? |
| **Communication & autonomy** | |
| Providers introduce self | During your time in the health facility did the doctors, nurses, or other health care providers introduce themselves to you when they first saw you? |
| Called appropriately | Did the doctors, nurses, or other health care providers call you by your preferred name (or in an appropriate way)? |
| Involved in care | Did you feel like the doctors, nurses, or other staff at the facility involved you in decisions about your care? |
| Explain exams | Did the doctors and nurses explain to you why they were doing examinations or procedures on you? |
| Explain medicines | Did the doctors and nurses explain to you why they were giving you any medicine? |
| Consent before procedures | Did the doctors, nurses or other staff at the facility ask your permission/consent before doing procedures and examinations on you? |
| Able to ask questions | Did you feel you could ask the doctors, nurses, or other staff at the facility any questions you had? |
| Language/spoke in a way you understood | Did the doctors, nurses or other staff at the facility speak to you in a language or at a level you could understand? |
| **Responsive & Supportive care** | |
| Wait time | How did you feel about the amount of time you waited? Would you say it was very short, just a little long, somewhat long, or very long? |
| Took best care | Did you feel the doctors, nurses, or other staff at the facility took the best care of you? |
| Trust providers | Did you feel you could completely trust the doctors, nurses, or other staff at the facility with regards to your care? |
| Felt safe | In general, did you feel safe in the health facility? |
| Bribes | During your time at the facility, did any staff at the facility ask you or your family for a bribe? |
| Ask about feeling | Did the doctors and nurses at the facility talk to you about how you were feeling? |
| Paid attention when help needed | When you needed help, did you feel the doctors, nurses, or other staff at the facility paid attention? |
| Enough staff | Do you think there was enough health staff in the facility to care for you? |
| Cleanliness | Thinking about the wards, washrooms, and the general environment of the health facility, will you say the facility was very clean, clean, dirty, or very dirty? |
| Notes: All questions have responses options 0. No, never; 1. Yes, a few times; 2. Yes, most of the time; 3. Yes, all the time, except the following:   - Wait time: 0. It was just right; 1. It was somewhat long; 2. It was very long; 3. It was extremely long - Introduction options: 0. No, none of them; 1. Yes, a few of them; 2. Yes, most of them; 3. Yes, all of them - Neglect, verbal, and physical abuse options: 0. No, never; 1. Yes, once; 2. Yes, a few times; 3. Yes, many times - Cleanliness 0. Very clean; 1. Clean; 2. Dirty; 3. Very dirty | |
